# Polysaccharides: potential bioactive macromolecules for Alzheimer’s disease

**DOI:** 10.3389/fnut.2023.1249018

**Published:** 2023-09-15

**Authors:** Gong Peng, Ming Li, Zhaoli Meng

**Affiliations:** ^1^Laboratory of Tumor Immunology, The First Hospital of Jilin University, Changchun, China; ^2^Department of Neurology, The Second Hospital of Nanchang University, Nanchang, China

**Keywords:** polysaccharide, Alzheimer’s disease, amyloid-beta, oxidative stress, inflammation

## Abstract

Alzheimer’s disease (AD) is one of the leading causes of death and disability. AD is a devastating disease that has caused an overwhelming burden. However, no disease-modified treatment was discovered. The approval of sodium oligomannate (GV-971) in mild-moderate AD patients has attracted great attention to investigate the role of saccharides in AD. Therefore, summarizing and explaining the role of saccharides in AD is urgent and promising. Recent studies showed that polysaccharides (PSs) potentially benefit AD *in vitro* and *in vivo*. PSs could alleviate the pathological damage and improve cognitive symptoms via (1) antagonizing the toxicity of abnormal amyloid-beta and tau proteins; (2) attenuating oxidative stress and proinflammation; (3) rebuilding neuroplasticity. PSs exhibit one-multiple pathological hits of AD. However, a thorough chemical investigation is needed for further study.

## 1. Introduction

Alzheimer’s disease (AD) is a devastating disease characterized by a deterioration of cognitive decline and loss of the ability to self-care (1, 2). AD is the most common type of dementia. According to the ‘Alzheimer’s disease facts and figures, 2023’, Alzheimer’s remains the fifth leading cause of death among Americans 65 years and older, the official death certificates recorded 121,499 deaths from AD in 2019 (3). Moreover, AD is the leading cause of death worldwide and places a heavy burden on communities and healthcare systems.

However, no disease-modified treatment is available currently, although AD has been introduced for centuries, and therapies aimed at amyloid-beta (Aβ) peptides have been investigated for decades. Fortunately, the approval of the sodium oligomannate (GV-971), an algae-derived acidic oligosaccharide, in individuals with mild-moderate AD by National Medical Products Administration has attracted great attention. Notably, concerns and doubts remained immense about the effectiveness of this primarily anti-neuroinflammatory drug (4). However, with caution and hope, polysaccharides (PSs) have shown their abilities against misfolded Aβ and tau proteins, restoring mitochondrial dysfunction, and proinflammation, and destroyed neuroplasticity in recent years (5–13).

Therefore, searching, summarizing, and exploring the anti-AD property of PSs is urgent and potentially promising. Furthermore, PSs are abundant in nature and there are many treasures to be probed. We will first introduce the current pathological hypothesis of AD and summarize the PSs targeting AD according to the source from which they are derived. In the end, we will discuss the potential gaps in propelling PSs to clinical use. With the introduction of this critical review, we will come to understand the potential role of PSs in the anti-AD and further prospecting.

## 2. Pathophysiology of AD

**Figure 1:**
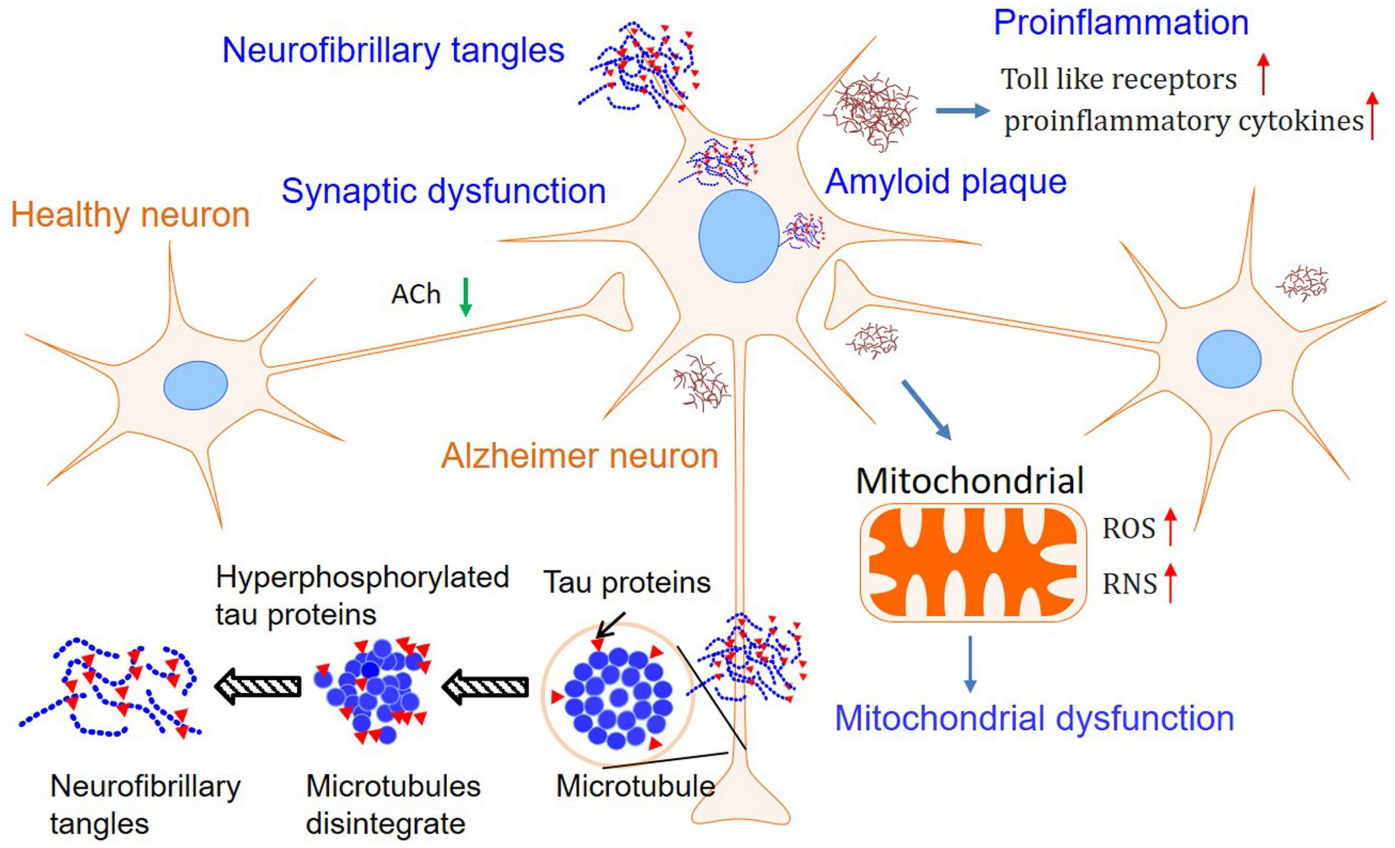
Mechanisms involved in Alzheimer’s disease. Although the Amyloid plaques, tau phosphorylation, and neurofibrillary tangles are still considered as the features of Alzheimer’s disease, hypotheses involved in mitochondrial dysfunction and pro-inflammation in neurons have attracted growing interest.

Although the first patient with AD was described more than one century ago, the precise mechanism of this devastating disease is currently unknown (1, 3). Based on the pathological findings, primarily amyloid plaques and neurofibrillary tangles, there are several hypotheses leading to the cause of AD. Among them, Aβ theory is the most sophisticated hypothesis and is followed by the tau proteins hypothesis. Furthermore, mitochondrial failure, proinflammation, and impaired neuroplasticity are assumed to deteriorate the pathological and clinical features (2). However, Aβ and tau are more likely to jointly initiate the essential pathological incidence, and other subsequent dysfunctions deteriorate the situation of the brain. Furthermore, metabolic disturbance and diet habits may also be involved in the processing of AD (1, 14–21). Finally, the mechanisms of AD are involved in abnormal Aβ and tau, mitochondrial failure, pro-inflammation, and impaired neuroplasticity, which were summarized in Figure 1. Therefore, drugs that target one of the steps in the progression of AD may induce multiple alterations in physiology (14–21). Herein, we will briefly introduce the hypothesis separately to facilitate the demonstration of the effects of PSs on AD.

### 2.1. Controversial Aβ hypothesis

Aβ is the main component of amyloid plaques in the central nervous system, which is believed to be the pathological marker of AD. Amyloid plaques were always associated with a decrease in proliferation and neurogenesis (15, 22). Aβ derived from the amyloid precursor protein (APP), which is cleaved by β-secretase beta-site amyloid precursor protein cleaving enzyme-1 (BACE-1) to yield APPβ and the Aβ is released after APPβ is cleaved by γ-secretase. The precise diagram of the processing of Aβ was illustrated by Querfurth and LaFerla (2). Aβ peptides are natural products of metabolism amino acids involved in neurogenesis, synaptic plasticity, and memory formation. Monomers of Aβ_40_ were much more prevalent than Aβ_42_, which was more prone to aggregation and neuron-toxicity. Furthermore, the ratio of Aβ_40_/Aβ_42_ was assumed to be more reliable for the formation of amyloid plaques (2, 19). Therefore, regardless of the physiological effect of Aβ, reducing the APP, inhibiting the BACE-1, or γ-secretase ideally reduces the level of Aβ and potentially mitigates the pathological dysfunction. However, most clinical trials of drugs targeting Aβ failed in recent years, raising concern about whether Aβ is the key factor in the progression of AD (23, 24). Fortunately, a new drug (lecanemab) targeting Aβ was approved by FDA most recently (25), regaining confidence in the hypothesis of Aβ in AD progression.

### 2.2. Tau hypothesis

Physiologically, tau protein modulates the microtubule and vesicle transport of neurons. Pathologically, hyperphosphorylated tau tends to aggregate and insoluble aggregation is the major component of the neurofibrillary tangles, and the tangle is another pathologic marker of AD (2, 21). The phosphorylation of tau proteins interacted with pathway molecules (2). Improper production and modification of tau protein resulted in microtubule disintegration, destroying the structure of the cell cytoskeleton and disrupting the neuron’s transport system. Furthermore, the ruined transport system led to cell malfunction and deficiency in neurotrophin. Elevated hyperphosphorylated tau and total tau closely were correlated with the severity of memory loss in individuals with AD (14, 26). Some research indicated that amyloid deposition and Aβ accumulation were the initial changes and the abnormal Aβ behaviors aroused dysfunctional tau. However, the cognitive condition was more closely correlated with tau homeostasis than Aβ (14, 26). Therefore, setting tau-target methods as a priority seems promising.

### 2.3. Mitochondrial failures

Mitochondrial failures were found both in individuals with AD and animal models (12, 27–31). Specifically, mitochondria failures included excessive oxidative stress, imbalance of fission and fusion, and mitophagy. Enhanced oxidative stress and the accumulation of reactive oxygen species (ROS) and reactive nitrogen species (RNS) were the most common features in AD (28). The mitochondrial dysfunction directly destroyed energy homeostasis, which sought to deteriorate AD. In the meantime, the excessive mitochondrial products impaired neurogenesis and disrupted metal homeostasis, synaptic plasticity, neurotransmission, and promoted mitochondrial apoptosis (30). Recent studies better illustrated the role of impaired mitochondria in AD. Beck et al. demonstrated that a specialized mitochondrial unfolded protein response was activated under the circumstance of excessive accumulation of misfolded protein in the AD brains (28). Furthermore, Adav and colleagues suggested that dysregulated mitochondrial complexes, including the electron transport chain (ETC) and ATP-synthase, devastated the brains (27). The intermittent oxidative product, (hydrogen peroxide) H_2_O_2_, has successfully created AD-like alterations in animal models (32, 33). Moreover, amyloid plaques and neurofibrillary tangles promoted mitochondrial dysfunctions and in turn, deteriorated misfolded Aβ and tau (29). Therefore, restoring the function of mitochondria will mitigate pathological damage and alleviate the symptoms of AD (34).

### 2.4. Proinflammation

Proinflammatory activities are observed in neurodegenerative diseases and AD. Misfolded and aggregated proteins bond to the pattern recognition receptors and spark the innate immune response (35). Activated microglia and reactive astrocytes surrounded and tried to tackle plaques at the beginning. However, the chronically activated microglia released too many inflammatory molecules and exacerbated the pathogenesis. Providing a transcriptomic framework for microglial activation, genes within homeostatic microglia represented a high risk of AD, suggesting early microglial dysregulation in AD. Pro-inflammatory factors are positively correlated with neuropathology and cognitive decline (20). Moreover, anti-inflammatory phagocytic microglia cells prevented the Aβ seeding (22). Therefore, microglia had contradictory roles in AD pathogenesis. In detail, microglia degraded misfolded proteins and released excessive pro-inflammatory factors, and these changes reconfirmed the transcriptomic study (20). Therefore, promoting pro-inflammatory and inhibiting anti-inflammatory activities within microglia by normalizing the regulators predicts a practicable therapeutic strategy.

### 2.5. Synaptic dysfunction

Synaptic failure is the predominant feature of AD, as this collapse directly jeopardizes memory formation and learning, leading to cognitive decline. The pro-aggregated Aβ peptides destroyed the synaptic plasticity by unbalancing long-term potentiation (LTP) and long-term depression (LTD). Moreover, misfolded tau proteins synergistically disrupted the normal synaptic transmission and suppressed receptors which should facilitate memory formation (2). The receptors for N-methyl-d-aspartate (NMDA) and α-amino-3-hydroxy-5-methyl-4-isoxazole propionic acid (AMPA) were broken down and the function of nerve growth factors was compromised (2). The signaling pathway of nicotinic acetylcholine (ACh) receptor and the release of ACh from the presynaptic terminal were impaired in AD. Therefore, an increase in the supply of ACh or a decrease in consumption of ACh by using acetylcholinesterase inhibitors (AChEIs) improved memory loss, although the effect is mild (18). The lower efficiency of ACh supplement is partially due to the blood–brain barrier or ACh alone does not reverse synaptic failure in AD (36). Modified tools for drug delivery are critical for AD treatment.

## 3. Polysaccharides targeting AD

**Figure 2:**
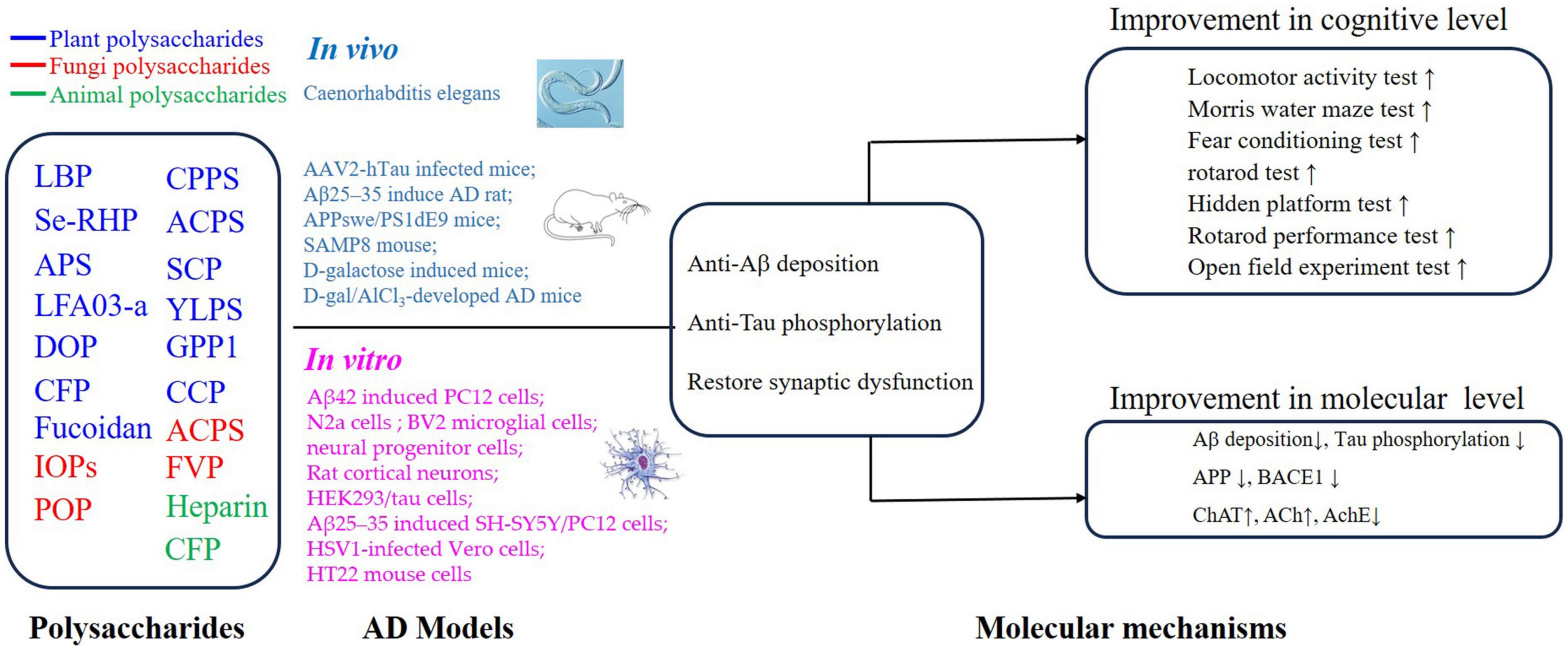
Mechanisms of polysaccharides on the improvement of cognitive impairment in AD via regulation of Aβ deposition, Tau phosphorylation, and synaptic dysfunction.

**Figure 3:**
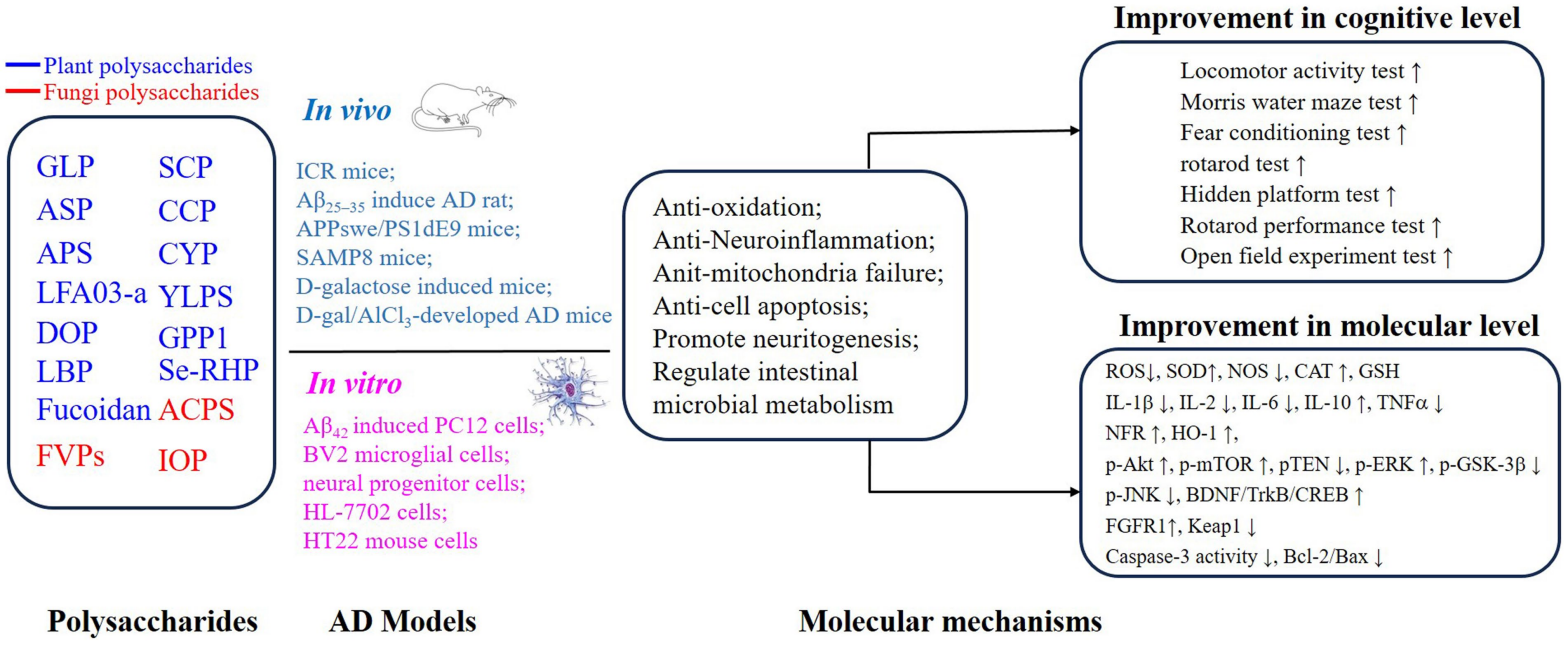
Mechanisms of polysaccharides improving the cognitive function on AD through neuroprotective effects.

PSs have been widely investigated because of their excellent safety for humans and well-established advantages in antioxidation and inflammatory regulation (5, 29, 37–40). Importantly, the approval of a drug, which originated from oligomannate, in mild-moderate AD patients has raised great interest in exploring natural products against aging. PSs are long chains of carbohydrate molecules and monosaccharides, which are bound together with glycosidic linkage. Structurally, PSs were classified into 3 types (linear, lowly branched, and highly branched PSs) according to the amounts of branches. In terms of composition, they are polymerized by homo-monoses or heter-monoses, which are named homopolysaccharides or heteropolysaccharides (41, 42). Regarding the source, PSs can be purified from plants, fungi, and animals, and their bioactivities were progressively studied due to being less toxic (38, 41, 43). Nowadays, many PSs show their anti-AD effects *in vivo* or *in vitro*, and the underlying mechanisms targeting AD were investigated. As shown in Figures 2, 3, we will introduce the potential PSs in AD treatments and summarize their structure and mechanisms according to the sources from which they are purified.

### 3.1. Plant polysaccharides

Plants are the most abundant sources of PSs. Plant PSs target the processing, aggregation, and clearance of misfolded A peptides and tau proteins (10, 11, 37, 40, 44–47). In addition, these PSs can regulate oxidative stress and inflammatory dysfunctions (5, 10, 12, 29, 31, 32, 39, 44, 47–56). In special, PSs from Chinese traditional medicinal crops including *Angelica*, *Codonopsis pilosula*, *Polygonatum sibiricum*, and *Pyropia haitanensis* have the potential to modulate neuroplasticity, promote neurogenesis, and normalize neurotransmission (5, 38, 39, 56).

#### 3.1.1. The structure feature of anti-AD PSs from plants

The activity of PSs is related to their structure features including molecular weight (MW), monosaccharide composition, glycosidic-linkage composition, conformation, and functional groups. However, several anti-AD polysaccharides have not been isolated and further purified. Hence, we could just discuss the anti-AD PSs whose structures were identified.

The anti-AD PSs from plants showed different ranges of MW and diverse monosaccharide compositions (Table 1). The MW of PSs extracted from the root of *Astragalus membranaceus* ranges from 3.96 to more than 2676.00 kDa, and the MW of most plant polysaccharides is distributed in 10 to 100 kDa. Such data indicated that MW is not an exclusive feature related to the anti-AD activity. The monosaccharide compositions of the plant polysaccharides were very complicated. Man, Gal, Glc, Fuc, Fru, Rha, Ara, GalA, and Xyl were detected among the plant PSs targeting AD, and Glc was the most common monosaccharide.

**Table 1:** Characteristic information, AD models and targeting mechanisms of anti-AD PSs.

Name	Source	Extraction methods	MW/kDa	Monosaccharide composition	AD targets	Activity	References
ASPs	Root of *Angelica sinensis*	Water extraction	NM	NM	Aβ, PIF, SDF	Ameliorate spatial learning and memory deficiency in Aβ_25-35_-induced AD rats.	(5)
CPPs	Root of *Codonopsis pilosula*	Water extraction	NM	NM	Tau, SDF	Attenuated AD-like cognitive impairments; Reduced Tau phosphorylation in hippocampus of AAV2-hTau infected mice.	(38)
LBPs	Fruit of *Lycium barbarum*	Water extraction	NM	NM	Aβ, Tau, MT	Inhibited apoptosis in H_2_O_2_ or CoCl_2_-treated PCl2 cells *in vitro*	(32, 40, 45)
Se-RHPs	Root of *Radix hedysari*	Water extraction	27.7–62.7	NM	Aβ, MT	Showed neuroprotective effects against Aβ_25–35_ induced oxidative stress SH-SY5Y human neuroblastoma cells.	(31)
PS-WNP	Rhizome of *Polygonatum sibiricum*	Water extraction	76	Man: Gal = 5.4: 12.1	MT, SDF	Protective effects against apoptosis in Aβ_25–35_ induced PC12 cells	(39)
CCP	Rhizome of *Coptis chinensis*	Water extraction	3.96	Glc	Aβ, MT	Showed the neuroprotective effects in Aβ_1–42_ transgenic CL4176 *Caenorhabditis elegans*	(29, 37)
APSs	Root of *Astragalus membranaceus*	Water extraction	0.86–2676.00	Fuc: myo-inositol: Fru: sorbitol: Glc = 1: 1.4: 2.1: 13.7: 91.5	PIF	Ameliorated the AD pathlogy in HFstz-augmented activation of APPswe/PS1dE9 mice	(53)
GPP1	*Gynostemma pentaphyllum* body	Water extraction	NM	Glc: Man: Gal: Rra: Ara = 10.0: 8.0:6.2: 1.5: 1.5	MT	Protected PC12 cells from Aβ_25–35_ induced cytotoxicity	(12)
LJW0F2	Flower of *Lonicera japonica Thunb*	Water extraction	37.1	Gulcose = 99.7%	Aβ	Attenuated the cytotoxicity induced by Aβ_42_ aggregation in SH-SY5Y neuroblastoma cells	(46)
LFA03-a	Flower of *Lonicera japonica Thunb*	Water extraction	67	Rha: Ara: Gal: GalA = 18.1: 25.3: 36.8: 19.5	Aβ, PIF	Inhibited Aβ_42_ aggregation in PC12 cells and promoted neuritogenesis	(10)
VTPs	Grape stalk of *Vitis vinifera L.*	Water extraction	19–40	Rha, Ara, Xyl, Man, Gal, and Glc	PIF	Reduced the toxic effects of Aβ_25–35_ on neurons in AD rats	(57)
DOP	Stem of *Dendrobium officinale*	Water extraction	NM	NM	Aβ, Tau, PIF	The protective effect of DOPs on hippocampal neuron;	(44, 54)
						Improved learning and memory disability in SAMP8 mice and in ovariectomy-and D-galactose-induced mice.	
SCP	Fruit of *Schisandra Chinensis*	Water extraction	NM	Man, Rha, GlcA, Glc, Gal, and Ara	Aβ, Tau, MT, PIF, SDF	Improved the cognition and histopathological changes of Aβ_25-35_-induced AD mice;	(11, 47)
						Reduced the deposition of Aβ; Activated the glial cells in the hippocampus;	
						Decreased Aβ and p-tau expressions;	
						Reduced the level of hippocampal NOS,	
						GSK-3β, ACh E, and increased SOD activity in hippocampus of Aβ_1-42_-induced AD rats.	
CYP	Root of *Corydalis yanhusuo*	Water extraction	75.5	mainly D-Glc	MT	Protected PC12 cells against Aβ_25–35_ induced toxicity	(52)
YLPS	Root of *Yulangsan*	Water extraction	14.301	Glc: Ara = 90.8: 9.2	MT, PIF	Protective effect suppressing aging in D-galactose induced mice	(51)
Brown algal PSs	Brown algal	Uronic acid extraction	NM	Rha, Fuc, Ara, Xyl, Man, Gal, Glc	Aβ, Tau	Prevent the HSV1-induced accumulation of the characteristic abnormal molecules of AD -like brains containing P-tau and β-amyloid in Vero cells.	(58)
Fucoidan	Several species of brown seaweed	Water extraction	266.1	NM	Aβ, MT, SDF	Decreased Aβ accumulation and ROS production in a transgenic *Caenorhabditis elegans* AD model	(55, 56)
SFPS	*Sargassum fusiforme algal*	Water extraction	75	Glc: GlcA: Xyl: Rha: Man: ManA: Gal: Fuc and GluA = 1: 3.8: 5.2: 5.3: 6.1: 8.8: 9.1: 28.9: 38.9	MT	Decelerated aging process in HL-7702 cells and D-galactose-induced ICR mice	(50)
Porphyrin P1	Red alga *Pyropia haitanensis*	Water extraction	29.7	NM	Aβ, PIF	Ameliorated the learning and memory impairment in Aβ_1–40_-induced AD mice.	(59)
ACPS	Body of *Amanita caesarea*	Water extraction	18.6, 33.5	Xyl, Man, Gal and Glc	Aβ, MT, SDF	Improved the functional of central cholinergic system and reduced acetylcholine esterase levels, and inhibited the apoptosis of HT22 cells induced by L-Glu.	(60)
IOPS	Body of *Inonotus obliquus*	Water extraction	111.9	Ara, Rha, Fuc, Xyl, Man, Gal, Glc, GlcA, GalA	Aβ, Tau, MT	Improved the pathological behaviors related to memory and cognition, Reduced the deposition of β-amyloid peptides and oxidative stress enzymes in APP/PS1 mice.	(61)
FVP			54.78	Man, Ribose, Glc, galactose, xylose = 4.07:4.54:3.07:1:2.2168	MT, PIF, SDF	Improved scopolamine-induced impairment of learning and memory in D-galactose-induced rats and in mice.	(62, 63)
FVP-1	Body of *Flammulina velutipes*		28	Glu: Fuc: Man: Gal = 81.3%:3.0%:3.6%:12.%	MT, PIF, SDF	Down-regulated Aβ-induced inflammation in BV2 cells, and inhibited Aβ-induced phagocytosis in microglia;	(48, 64, 65)
FVP-2	Distilled water by ultrasonic treatment		26	Glu: Fuc: Xyl: Man: Gal = 57.9%: 5.5%:9.5%:15.1%:12.%		Promoted neural progenitor cell proliferation to enhance neurogenesis and alleviated cognitive deficits in APP/PS1 mice.	
GLP-I			15.0	Ara, Rha, Xyl, Man, Glc = 4.66: 1.23: 3.14: 0.61: 1.29			
GLP-II	Body of *Ganoderma lucidum*	Water extraction		Ara, Xyl, Glc = 2.82: 1.33: 0.87			
GLP-III				Ara, Rha, Xyl, Gla: Man, Glc = 5.09:0.52:1.07:1.29:0.48:2.76			
GLP-IV				Ara, Rha, Fuc, Xyl: Man, Glc = 4.73: 0.65: 0.72: 2.27: 0.52: 0.92			
CFP	Body of *Cucumaria Frondosa*	Acetone	26.9		Aβ	inhibit the aggregation of Aβ40 and disassemble mature fibrils; Reduced the cytotoxicity of Aβ in N2a cells	(66)
POP	Body of *Pleurotus ostreatus*	Ultrasonic-assisted extraction	NM	NM	Aβ, Tau, MT, SDF	Decreased escape latency and increased crossing parameters of platform quadrant in a Morris water maze test in AlCl_3_-induced cognitive mice.	(7)
Enoxaparin	Chemical synthesis		3.8–5.0	NM	Aβ	Improved cognition in APPswe/PS1dE9 mice; reduced β-amyloid load in APP23 transgenic mice.	(67, 68)
COS	Chitin derivative		1	NM	Aβ	Reduced the aggregation and cytotoxicity of Aβ_40_	(69)

#### 3.1.2. The mechanism targeting AD of PSs from plants

In Aβ or P-Tau-induced models *in vivo*, the plant PSs including ASPs, CPPs, VTPs, and SCP significantly improved the cognitive, memory, and learning disorders through behavior tests. However, the effects of these PSs on AD and their underlying mechanisms were considered to be different. Du et al. showed that ASP could increase Ach levels and decrease AChE levels, which can improve cholinergic function. Furthermore, ASP extracted from the root of *Angelica sinensis* decreased free radical metabolites and inhibited the expression of pro-inflammatory factors. Specifically, ASP activated superoxide dismutase (SOD) and catalase (CAT) activity and suppressed malondialdehyde (MDA) activity, restoring impaired mitochondrial function in AD. Moreover, ASP protected the hippocampal neurons from the toxicity of Aβ. In addition, ASP could eliminate the toxicity of Aβ by activating the BDNF/TrkB/CREB pathway in the hippocampus of rats (5). CPPs prevented hyperphosphorylation of tau and alleviated cognitive impairments in hTau-expressing mice. Zhang reported that intragastric administration of CPPs reduced phosphorylation of Tau at Ser199, Ser202/Thr205, and Thr231 in the hippocampus. Furthermore, CPPs restored synaptic plasticity and synaptogenesis by increasing the expression of synaptotagmin and synaptophysin (38). Thus, CPPs administration prevented poor cognitive performance induced by hTau. Besides, VTP suppressed the pro-inflammatory factors in AD rats ameliorating the cognitive malfunction by decreasing the level of pro-inflammatory factors in a dose-dependent manner via regulating nuclear factor kappa B/inhibitor kappa B alpha (57). Therefore, the regulation of the VTPs on the inflammation was impacted by the NFκB/IκB-α pathway. The SCP from the fruit of *Schisandra Chinensis* could reduce the phosphorylated tau protein, the aggregation of Aβ, and oxidative damage and could reverse changes in neurotransmitters in the AD rats (29). Moreover, the SCP repaired the neuron loss and promoted neurogenesis and synaptic transmission (29). Furthermore, the SCP also performed an anti-inflammatory function relying on the activation of the NF-κB/MAPK pathway (47). Porphyran, named P1, an antioxidant polysaccharide isolated from the red algae *Pyropia haitanensis*, reduced the cytotoxicity of Aβ peptides. P1 decreased the activities of oxygenated free radicals. Furthermore, P1 enhanced ChAT activity and suppressed AChE activity in the Aβ_1–40_-induced AD mice model. Therefore, alleviation of cognitive decline due to increased anti-oxidant activity and Ach metabolism (59). Moreover, LP-G2, a sulfated galactan, isolated and purified from *Pyropia haitanensis*, performed anti-complementary activity. LP-G2 is potentially targeted to the pro-inflammatory activity that was shared in AD individuals.

In APP/PS1 mice, the effects of APSs and IOPS on AD were investigated. In the APPswe/PS1∆E9 (APP/PS1) transgenic mice administrated with a combination of a high-fat diet and a low-dose injection of streptozotocin, the APSs suppressed metabolic-activated plaque-associated astrocytes and microglia and improved the performance of nest construction in the transgenic AD model (53). IOPS also reduced the aggregation of Aβ peptides and tau proteins in the brains of APP/PS1 mice. Briefly, the IOPS took advantage mainly by modulating mitochondrial apoptosis and oxidative stress through the Nrf2 pathway.

In D-galactose-induced rats or mice, the PSs including DOP, YLPS, and SFPS improved learning and memory disability. Pretreatment with DOP could reduce the accumulation of the hippocampal Aβ_42_ and phosphorylated tau proteins, which was initiated and deteriorated by microglial activation. Moreover, DOP alleviated cognitive decline mainly via regulating microglial activity, attenuated oxidative stress, and reduced neuro-inflammation via the up-regulating Nrf2/HO-1 pathway (70). Root of Yulangsan PSs, YLPS, greatly improved the general condition of the mice and significantly increased the levels of antioxidant enzymes, which was demonstrated as an anti-oxidant feature. Furthermore, YLPS promoted the expression of anti-inflammatory factors and increased the expression of anti-inflammatory cytokines. *Sargassum fusiforme* polysaccharides (SFPS) exerted antioxidant activity by activating the Nrf2 pathway. SFPS reversed D-galactose-induced antioxidant defense and boosted mitochondrial integrity (50). SFPS65A was also isolated from a brown alga (*S. fusiforme*), Hu and colleagues revealed that SFPS65A ameliorates learning and memory defects in scopolamine-, ethanol-, and sodium nitrite-treated mice (54).

Fucoidan, a complex sulfated PSs, attenuated the Aβ_25–35_ and D-Gal-induced neurotoxicity in mice and transgenic *Caenorhabditis elegans* (55, 56). Another fucoidan polysaccharide sulfate reduced the Aβ-induced toxicity by reducing the accumulation of Aβ and decreasing the level of ROS in transgenic *Caenorhabditis elegans*. Furthermore, FPS reduced excessive ROS and facilitated clearance of the Aβ by increasing proteasome activity (55). *Coptis chinensis* polysaccharide (CCP) exerted neuroprotective effects on Aβ-induced toxicity in a transgenic *Caenorhabditis elegans* model of AD by increasing the expression levels of HSP (29, 37).

Besides the study of plant PSs targeting AD *in vivo*, numerous plant PSs also displayed significant anti-AD activity in cell models. In a combination of Aβ_25–35_- and D-galactose-induced PC12 cell model of AD, Wei et al. indicated that fucoidan mitigated mitochondrial dysfunction by decreasing caspase activity and expression of apoptosis proteins. Furthermore, the fucoidan restored ACh and choline acetyltransferase (ChAT) and repressed the activity of acetylcholine esterase (AChE). Moreover, fucoidan increased the expression of SOD and glutathione (GSH) (56). Therefore, fucoidan exerted the protective effect via reducing oxidative stress, restoring the cholinergic system, and inhibiting the caspase-dependent apoptosis pathway (56). LBPs from the fruit of *Lycium barbarum* protected against Aβ peptide and homocysteine-induced neurotoxicity in rat cortical neurons. Yu and colleagues suggested that LBP could protect against the toxicity of Aβ-peptide by reducing the level of caspase-3, which was activated by Aβ-peptide. Furthermore, LBPs also reduced the Aβ-induced-phosphorylation of JNK-1 (Thr183/Tyr185) and its substrates (c-Jun-I and c-Jun-II) (40). In addition, the protective effect of LBPs was presented in a dose-dependent manner (40). On the other hand, Ho et al. insisted that LBP had a protective function against homocysteine (Hcy)-induced tau hyperphosphorylation (45). Specifically, LBPs attenuated Hcy-induced neuronal cell death and apoptosis in primary cortical neurons, suppressed Hcy-induced multiple phosphorylation sites of tau, and alleviated Hcy-induced tau cleavage (45). Therefore, LBPs had the potential to primarily target Aβ- and tau-induced poor consequences in neuronal cells. Additionally, LBPs perhaps benefited H_2_O_2_-treated PC12 cells and CoCl_2_-treated rats, which mimicked partial characteristics of AD *in vitro*, by inhibiting excessive oxidative stress (32). Cao et al. revealed that one type of LBPs could reverse the increase in free radical metabolites induced by H_2_O_2_ and mitigate mitochondrial damage. Furthermore, LBP reduced caspase-3 and caspase-9 activity by regulating the Nrf2 pathway (32). Additionally, LBPs perhaps benefited H_2_O_2_-treated PC12 cells and CoCl_2_-treated rats, which mimicked partial characteristics of AD *in vitro* and *in vivo*, by inhibiting excessive oxidative stress (32). *Radix hedysari* polysaccharides (RHPs) are extracted from the roots of Radix *hedysari*, and Selenium *Radix hedysari* polysaccharides (Se-RHPs) are the selenylation derivatives of RHPs. Se-RHPs had a favorable effect against oxidative stress and apoptosis in Aβ_25–35_-treated SH-SY5Y cells and reduced the activity of superoxide radicals, hydroxyl radicals, and diphenylpicrylhydrazine (DPPH-H) radicals. Meanwhile, Se-RHPs and RHPs prevented SH-SY5Y cells from oxidative stress and apoptosis in Aβ_25–35_ treated cells (31). A polysaccharide (PS-WNP) from *Polygonatum sibiricum* attenuated the Aβ-induced neurotoxicity in PC12 cells. Zhang et al. demonstrated that pretreatment with PS-WNP can significantly reduce the apoptosis and the Bax/Bcl-2 ratio, restoring mitochondrial function in Aβ_25–35_ treated PC12 cells. Additionally, PS-WNP decreased the caspase-3 level and increased the protein level of phosphorylated Akt (p-Akt) in PC12 cells (39). *Gynostemma pentaphyllum* polysaccharide (GPP) protected P12 cells from Aβ_25–35_-induced apoptosis and mitochondrial dysfunction. Functional analysis revealed that GPP1 reduced the Aβ_25–35_-induced cytotoxicity, DNA damage, free radical metabolism, and pro-apoptosis-associated proteins (12). Moreover, GPP1 also reduced the ratio of Bax/Bcl-2 protein and caspase-3 activity. Therefore, GPP1 inhibited oxidative stress and suppressed the mitochondrial apoptotic pathway to confront Aβ_25–35_-induced damage (12). LJW0F2, a glucan isolated from flowers of *Lonicera japonica* Thunb, inhibited the aggregation and neurotoxicity of Aβ_42_. In SH-SY5Y neuroblastoma cell models, LJW0F2 inhibited the Aβ_42_ aggregation in a dose-dependent manner and ameliorated the cytotoxicity induced by Aβ_42_ aggregation (46). Additionally, LFA03-a, a pectin isolated from *Lonicera japonica* Thunb, blocked the Aβ_42_ aggregation in a dose-dependent manner and promoted neurogenesis. Furthermore, LFA03-a inhibited Aβ_42_ oligomerization and fibril formation. Of note, LFA03-a could promote the differentiation and neurogenesis of PC12 cells (10). *Corydalis yanhusuo* PSs (CYPs) showed a protective role in PC12 cells with Aβ_25–35_-induced cytotoxicity, apoptosis, and mitochondrial dysfunction. CYPs eased cell death, DNA fragmentation, mitochondrial dysfunction, and release of mitochondrial cytochrome c and lactate dehydrogenase (LDH) in the AD cell model (52). CYPs decreased the Bax/Bcl2 ratio as well as the expression of cleaved caspase-8, caspase-9, and caspase-3. Therefore, CYPs inhibited cell apoptosis by regulating mitochondrial apoptotic and death receptor pathway (52). *Brown algal* polysaccharides are isolated from the *brown algal*, the fucans prevented the accumulation of herpes simplex virus type 1 (HSV1)-induced Aβ peptide and AD-like tau in Vero cells infected with HSV1 (58). Furthermore, CCP restored Aβ-induced mitochondrial dysfunction and inhibited cell apoptosis in PC12 cells. Briefly, CCP attenuated the consequences of cell death, lactate dehydrogenase (LDH) release, nuclear fragmentation, mitochondrial dysfunction, and release of cytochrome c from mitochondria in Aβ_25–35_-expressing PC12 cells (29). Regarding the signaling pathway, CCP activated the c-Jun N-terminal kinase (JNK), and decreased the Bax/Bcl2 ratio and cleaved caspase-3 (29). Therefore, CCP potentially benefited AD by recovering Aβ peptides-damaged mitochondrial function and regulating the JNK-dependent apoptotic pathway (29, 37).

### 3.2. Fungal PSs

Not like the most widely used isolation methods for PSs from plants, multiple methods have been applied to extract PSs from fungi, including ultrasonic-assisted extraction and solvent extraction methods (60, 71). These fungal PSs were different in the structure features including molecular weight and monosaccharide composition (Table 1). PSs isolated from *Ganoderma lucidum*, *Flammulina velutipes*, *Pleurotus ostreatus*, *Amanita caesarea*, and *Inonotus obliquus* protected against the aging consequences of AD. Specifically, it is suggested that fungal PSs benefit AD a lot, including reducing the aggregation of Aβ peptides and tau proteins (7, 60, 61, 71), eliminating excessive oxidative stress (7, 60–62, 71), pro-inflammatory (41, 48), and restoring dysregulated neuroplasticity (7, 60, 62, 64, 71) (Figures 2, 3).

#### 3.2.1. The structure feature of anti-AD PSs from fungus

The MW of the fungi polysaccharides in Table 1 ranges from 15 to 111.9 kDa. Three polysaccharides FVP, FVP-1, and FVP-2 were extracted from *Flammulina velutipes* bodies (62, 72). The MW of FVP, FVP-1, and FVP-2 were 54.78, 28, and 268 kDa, respectively. In addition, four polysaccharide components were isolated from *Ganoderma lucidum*, they also have different MW and monosaccharide compositions (65).

#### 3.2.2. The mechanism targeting AD of fungal PSs

In APP/PS1 mice, IOPs and GLPs showed the potential to inhibit AD. IOPS is isolated from *Inonotus obliquus* bodies, Han et al. suggested that the IOPS had the potential to prevent oxidative stress and apoptosis in L-glutamic acid-damaged HT22 cells. The IOPS obtained an MW of 111.9 kDa, and the benefits of this IOPS possibly originated from the enhancement of Nrf2 signaling. The IOPS endorsed the anti-oxidative enzymes and associated proteins, while suppressing the pro-oxidative enzymes and proteins (61). Furthermore, IOPS also reduced the aggregation of Aβ peptides and tau proteins in the brains of APP/PS1 mice. Briefly, the IOPS took advantage mainly by modulating mitochondrial apoptosis and oxidative stress through the Nrf2 pathway (61). GLP from *Ganoderma lucidum* ameliorated microglia-mediated neuroinflammation and promoted proliferation of neural progenitor (48, 64). The MW of the GLP was about 15 kDa. Lipopolysaccharides (LPS) and Aβ_42_ granted the microglia activation. GLP decreased pro-inflammatory factors and increased expression of anti-inflammatory cytokine. GLP also suppressed microglia migration, morphological changes, and phagocytosis probabilities. Furthermore, the modulation of microglial activation of GLP was associated with expressions of MCP-1 and C1q (48). Additionally, GLP promoted neurogenesis and ameliorated cognitive decline in APP/PS1 transgenic mice. GLP increased the expression of the fibroblast growth factor receptor 1 (FGFR1) and promoted extracellular signal-regulated kinase (ERK) and AKT signaling pathways. GLP also suppressed microglia migration, morphological changes, and phagocytosis probabilities. Furthermore, the modulation of microglial activation of GLP was associated with expressions of MCP-1 and C1q (48).

In the rats with scopolamine-induced impairment of learning and memory, *Flammulina velutipes* polysaccharides (FVP) significantly improved cognitive performance. The benefits of FVP partially came from the elevated SOD and GSH-Px activity. Furthermore, FVP enhanced the activities of Ach and ChAT and suppressed the activity of AChE. Moreover, the FVP also modulated the composition of gut microbiota to facilitate the protection in AD models (63). The FVP indirectly resulted in the increase of the anti-inflammatory mediators that closely interacted with the alteration of the gut microbiota (63). Therefore, FVP featured antioxidant, anti-inflammatory, and pro-cholinergic effects in the rat AD models (63). What is more, the protective effects of FVP could be enhanced by compatibilization with ginsenosides (62). *In vitro* and in D-galactose-induced aging mice, the conventional water extracted-*Flammulina velutipes* PSs (FPS) and sulfated FPS (SFPS) exhibited antioxidant and anti-ageing activities (41).

In an AD model induced with D-galactose and AlCl_3_, *Pleurotus ostreatus* PSs (POP) ameliorated cognitive defects. Specifically, POP decreased the escape latency in a Morris water maze test and improved the performance in the step-down test. Furthermore, POP promoted enzyme activities of antioxidation and improved the cholinergic function of central nervous (7, 73). Importantly, POP elevated the expression of protein phosphatase 2A and decreased the expression of APP and BACE1. In terms of the biochemical changes, POP inhibited aggregation of Aβ peptide and phosphorylation of tau. In short, POP attenuated the cognitive decline by reducing the production of Aβ and inhibiting the phosphorylation of tau. Furthermore, POP also promoted antioxidant activity and increased cholinergic function (7). *Amanita caesarea* PSs are used mainly against the excessive species of oxidative stress during mitochondrial failure (60, 71). Li et al. reported that *Amanita caesarea* aqueous extract (AC) improved cognitive impairment and memory loss in a D-galactose and AlCl_3_-developed AD mouse model, and reversed L-glutamic acid (L-Glu)-induced HT22 cell apoptosis, as well as attenuated free oxidative stress and restored mitochondrial function. Moreover, in cell models, AC polysaccharides promoted neurogenesis by increasing the activity of protein kinase B (Akt) and the mammalian target of rapamycin (mTOR) (71). Furthermore, AC inhibited the deposition of Aβ peptides and promoted central cholinergic system function in the animal model of AD. In short, the AC facilitated cognitive function by impeding the aggregation of Aβ, restoring mitochondrial function, reducing consequences of mitochondrial dysregulation, and promoting neuroplasticity and neurogenesis (71).

### 3.3. Animal PSs

#### 3.3.1. The structure feature of anti-AD PSs from animals

Heparin and enoxaparin, a low MW derivative of heparin, have been used clinically as anticoagulants. Heparin is produced by basophils and mast cells in animals. Heparin is a glycosaminoglycan and is composed of various sulfated repeating disaccharide units. The average MW of heparin ranges from 3 to 30 kDa and the average MW of enoxaparin ranges from 3.8 to 5.0 kDa (74). The chitosan oligosaccharide is a deacetylated form of the chitin derivative. Unlike heparin and enoxaparin, COS has a very low MW of 1 kDa. The sea cucumber is a popular food, and it has many bioactive molecules such as polysaccharides. A polysaccharide from sea cucumber *Acaudina molpadioides* was extracted, the MW of which is 26.9 kDa (66).

#### 3.3.2. The mechanism targeting AD of animal PSs

Evidence indicated that heparin and enoxaparin are able to protect against pathological devastation in an animal model. Bergamaschini et al. demonstrated that enoxaparin reduced the accumulation of Aβ peptides and the cortical concentration of the total Aβ_1–40_ peptide. Furthermore, enoxaparin reduced the number of activated astrocytes surrounding β-amyloid deposits in transgenic mice that overexpressed the human amyloid precursor protein (67). In addition, enoxaparin was able to ameliorate cognitive decline in APPswe/PS1dE9 mice. However, enoxaparin had different effects on the Aβ deposition in different stages. Briefly, enoxaparin treatment at an early stage decreased guanidine HCl-extracted Aβ levels, while the introduction of enoxaparin at a later stage enhanced the Aβ accumulation (68). Besides, Cui et al. revealed that heparin and enoxaparin had no significant effect on the level of total APP in primary cortical neurons of Tg2576 mice, but both inhibited sAPPα and Aβ secretion (43, 75). In addition, heparin decreased the expression of BACE1 and α-secretase (ADAM10) (43) and a low anticoagulant heparin oligosaccharide also showed an inhibitory effect on BACE-1 (76).

In contrast to the potential benefits derived from heparin, some research also indicated that amyloid plaque and tau deposition deteriorated when heparin or enoxaparin were introduced (75, 77, 78). *In vitro* study illustrated that a low concentration of heparin stimulated the recombinant human BACE-1, while a higher concentration suppressed the activity of BACE-1. Chromatography showed that pro domain was necessary for the stimulation effect of heparin. Therefore, due to the lack of pro domain, the mature enzyme lost the capacity to be stimulated by heparin (77). Furthermore, peripheral administration of enoxaparin deteriorated amyloid plaque load via the aggregation of Aβ_40_ and Aβ_42_ and significantly increased the ratio of Aβ_42_/Aβ_40_ (75). Moreover, heparin possibly increased tau deposition (78). Therefore, heparin and enoxaparin could benefit AD, primarily aimed at the production and clearance of the Aβ peptides, and then improved cognitive performance (43, 67, 68, 76).

*Cucumaria frondose* PSs (CFP) are isolated from edible sea cucumbers *Cucumaria frondose*. An *in vitro* study showed that CFP could inhibit the aggregation of Aβ_40_. Further study suggested that the disassembling mature fibrils is the main mechanism of how CFP reduces cytotoxicity of Aβ (66). Therefore, CFP could benefit AD, primarily aimed at the clearance of the Aβ peptides.

COS has been traditionally used as a drug delivery tool. However, recent research showed that it can directly interact with Aβ peptides, reducing the cytotoxicity of Aβ peptides (69, 79–81). COS could inhibit Aβ deposition and fibril formation in a dose-dependent manner. Furthermore, COS decreased the toxicity in Aβ_1–42_-induced rat cortical neurons (80). Furthermore, COS alleviated cognitive defects in Aβ_1–42_-induced rats. Studies showed that COS attenuated neuronal apoptosis and promoted pro-antioxidants. Moreover, COS decreased the level of pro-inflammatory factors (81). Finally, COS possibly reduced the production of Aβ by inhibiting BACE1 in HEK293 APPswe cells (79). In summary, COS can benefit AD by reducing the production and enhancing the clearance of Aβ peptides as well as attenuating neuronal apoptosis by regulating pro-inflammatory and pro-antioxidant factors (79–81).

### 3.4. Association of anti-AD effect and structure of PSs derived from different sources

As described above, polysaccharides derived from different sources shared similar mechanisms of anti-AD effect. Therefore, it is attractive to find the relationship between the mechanism of anti-AD effect and the structure of polysaccharides. Because of the complexity of the structure of polysaccharides, the studies of polysaccharides with well-defined molecular structures have been limited previously. With the development of the technology of isolation/characterization in polysaccharides in recent years, a mass of polysaccharides with accurate structure were isolated and studied in AD models. Although the precise mechanism between structure and anti-AD activity is unknown, there are some hints that link structure and activity in anti-AD studies.

MW is one of the most important feathers for biomacromolecules. In developing a potential drug derived from polysaccharides for AD, the delivery efficiency through the blood–brain barrier should be considered. Of note, the difference in extraction methods may affect the molecular weight of purified polysaccharides. For example, the molecular weight of FVPs isolated with different methods was different (62, 63). With respect to extraction methods, water extraction was usually applied in the isolation of plant polysaccharides, while sonification and acetone extraction were more frequently used in the isolation of fungi and animal polysaccharides. Regarding the delivery drug efficiency of the blood–brain barrier, polysaccharides with a small molecular weight showed anti-AD effect *in vivo* (29, 48, 51, 57, 62, 65, 82).

The glycosyl residue composition is another important feather of polysaccharides, which may affect Anti-AD activity directly. The polysaccharides which were mainly composed of glucose showed anti-AD activity through the reduction of Aβ deposition (37, 46), while polysaccharides with glycosyls of rhamnose/arabinose/galactose showed anti-AD activity through anti-inflammation (10, 57). Detailed information on each PSs is listed in Table 1.

## 4. Conclusions and perspectives

AD is the most common dementia without effective therapy. The effort to purchase the healing for AD patients has been largely unsuccessful (16). There are some possibilities that lead to this poor result. Firstly, although the deposition of Aβ peptides is a common pathological finding in the brains of patients and intracranial injection of Aβ peptides produced a cognitive decline in animals, if the animal models mimick the symptoms of AD is not clarified (1, 2, 15, 22). Furthermore, reducing the level of Aβ successfully ameliorated cognitive defects *in vitro* and *in vivo* studies (80, 81, 83). However, the hypothesis of the Aβ cascade was questioned because misfolded peptides seem to initiate but not deteriorate the symptoms of AD patients (21, 68). It is likely that tau proteins take the brunt of worsening cognitive performance and deserve more attention (14). Second, the Aβ hypothesis is the principal one for familial AD but not for sporadic AD. The mutant AβPP and PSEN in familial AD greatly increased the production of Aβ peptides but the mutations are not common in sporadic AD patients (1, 2). Additionally, sporadic AD accounted for more than 90% of total AD cases (1, 3). Third, strategies aimed at reducing production, and clearance of Aβ could eliminate the normal function of Aβ peptides (7, 76, 79). Compared to the reduction of total Aβ peptides, decreasing the level of Aβ_42_ may be more feasible because Aβ_42_ is preferably toxicity and aggregated, but not Aβ_40_ (2). However, targeting Aβ_42_ is not prevalent in current research. Therefore, focusing only on the special Aβ peptide may be difficult and less promising. However, due to the complex interaction between pathological progression, multiple targets may be more promising, including the increase of BDNF activity, enhancement of mitochondrial function, and attenuation of toxicity induced by tau protein or Aβ peptides (34, 36).

PSs are a group of carbohydrate macromolecules derived primarily from natural products. According to what we mentioned above, PSs could benefit AD by targeting one-to-multiple pathological tags (5, 10, 38, 58, 62, 64, 68, 71, 80). Briefly, PSs aimed at reducing Aβ peptides and tau proteins, promoting anti-inflammation/anti-oxidation, suppressing pro-information/oxidative stress, promoting neurogenesis, and enhancing neurotransmission (10, 37, 38, 40, 43, 45, 68, 78). The features and potential therapeutic targets of these PSs are listed in Table 1.

Firstly, in terms of Aβ hypothesis, PSs reduced the production of Aβ peptides by inhibiting the activity of BACE-1 and γ-secretase. PSs increased clearance of Aβ by promoting proteasome-dependent protein degradation. Furthermore, PSs reduced cytotoxicity induced by Aβ peptides (10, 12, 29, 37, 40, 43–45, 53, 68). Second, PSs both reduced the phosphorylation and total protein levels of tau. In addition, PSs alleviated the deposition of tau proteins. PSs also attenuated tau aggregation-reduced cognitive (38, 78). Third, and most prevalently, PSs increased the level of anti-oxidative species (SOD, GSH) and decreased the activity of free radical molecules, such as ROS. Furthermore, PSs mitigated the consequences of mitochondrial-dependent apoptosis partially via Nrf2/ HO-1 pathway (12, 29, 31–33). Fourthly, PSs suppressed microglial activation and migration. Although some PSs could enhance inflammatory activities in some non-AD models, PSs promoted the expression of anti-inflammatory factors and decreased the level of inflammatory cytokines in AD models (44, 48, 53, 57). Finally, PSs enhanced central nervous cholinergic activities by encouraging the expression of ChAT and deactivating the AChE. Furthermore, PSs modulated neurogenesis via increasing the expression of BDNF and other neurotrophin (5, 11, 44, 56, 60, 64, 71).

Concerning PSs, chemical and structural information is in shortage. The chemical composition, structure, and function of PSs are much different when different procedures are applied to obtain PSs (42, 84–86). Specifically, isolation, deproteinization, separation, and purification have an impact on chemical composition and even inversely change the structure and activity. It is noteworthy that extraction methods are the most important factors that influence the structure and activity of PSs (42, 84, 85). Particularly, even an extraction temperature would result in a great discrepancy. Therefore, even derived from the same plants or fungi, contents of the PSs are heterogeneous (32, 40, 41, 45, 62). The results of studies aimed at exploring the potential therapeutic effects of PSs on AD were of great diversity (5, 38, 71). In summary, unthorough chemical and structural information, diverse extraction and purification methods, and various animal models in current studies limit the drug development of PSs in AD.

Unlike drug therapy for other diseases, the treatment of AD requires drugs in the blood to infiltrate through the blood–brain barrier (BBB). BBB is a continuous endothelial membrane within brain microvessels that has sealed cell-to-cell contacts, and is sheathed by mural vascular cells and perivascular astrocyte end-feet. The integrity of BBB is crucial for tight control of the chemical composition of brain interstitial fluid, which is critical for proper synaptic functioning, information processing, and neuronal connectivity (87). In the review, many PSs displayed their anti-AD activity by regulating Aβ and phosphorylation of tau protein levels in the mouse or rat models. However, the mechanism of crossing through BBB was not fully clear. The MW of anti-AD PSs ranges from 1 kDa to 2,676 kDa (53, 69), the low-MW polysaccharides might cross the BBB and regulate the neurons. In addition, the BBB breakdown in AD patients might affect the permeability in the hippocampus in the individuals, which might increase the possibility of the polysaccharides entering BBB (88). Moreover, multiple experiments revealed gut microbial dysbiosis leads to neurodegeneration and ultimately causes AD (89). As reported, regulation of the gut microbiota could attenuate the symptoms of AD via the gut-brain axis (90). Though oral administration with PSs contributed to alleviating the gut microbial dysbiosis, however, the effect of the anti-AD PSs on the gut-brain axis needs to be further studied.

In conclusion, PSs alleviated pathological damages and improved cognitive symptoms via (1) antagonizing the toxicity of abnormal Aβ and tau proteins; (2) attenuating oxidative stress and proinflammation; (3) improving neurogenesis and neuroplasticity (Figures 2, 3).

## Author contributions

ZM: conceptualization. GP and ML: writing – review and editing. ZM and GP: funding acquisition. All authors contributed to the article and approved the submitted version.

## Funding

This research was supported by the Project of Education Department of Jilin Province (JJKH20221049KJ; JJKH20221054KJ).

## Conflict of interest

The authors declare that the research was conducted in the absence of any commercial or financial relationships that could be construed as a potential conflict of interest.

## Publisher’s note

All claims expressed in this article are solely those of the authors and do not necessarily represent those of their affiliated organizations, or those of the publisher, the editors and the reviewers. Any product that may be evaluated in this article, or claim that may be made by its manufacturer, is not guaranteed or endorsed by the publisher.

## Figures and Tables

**Table:** 

AD	Alzheimer’s disease
PSs	Polysaccharides
APP	Amyloid precursor protein
Aβ	Amyloid-beta
BACE-1	beta-site amyloid precursor protein cleaving enzyme-1
ROS	Reactive oxygen species
ETC	Electron transport chain
Ach	Nicotinic acetylcholine
NMDA	N-methyl-d-aspartate
AMPA	α-amino-3-hydroxy-5-methyl-4-isoxazole propionic acid
AChEIs	Acetylcholinesterase inhibitors
hTau	human full-length tau
HSP	Heat shock protein
Nrf2	Nuclear factor erythroid 2-related factor 2
SAMP8	Senescence-accelerated mouse prone 8
JNK	c-Jun N-terminal kinase; Lipopolysaccharides
LDH	Lactate dehydrogenase
BBB	Blood–brain barrier
AChE	Acetylcholine esterase
GSH	Glutathione
MW	Molecular weight
Fru	Fructose
Suc	Sucrose
Lac	Lactose
Ara	Arabinose
Glc	Glucose
Gal	Galactose
Man	Mannose
Rha	Rhamnose
Xyl	Xylose
Fuc	Fucose
GlcA	Glucuronic acid
D-GlcN	Glucosamine
GlcNAc	N-acetylglucosamine
GalA	Glucuronic acid
ManA	Mannuronic acid
GluA	Guluronic acid
IDuA	L-Iduronic acid
NM	Not mentioned in the reference.
Tau	Tau hypothesis
MF	Mitochondrial failure
PIF	Proinflammation
SDF	Synaptic dysfunction
SOD	Superoxide dismutase
NOS	Nitric oxide synthase
CAT	Catalase
GSK-3β	Synthase kinase-3β
IL-1β	Interleukin-1β
IL-6	Interleukin-6
TNF-α	Tumor necrosis factor-α
IL-10	Interleukin-10
HO-1	Heme Oxygenase-1
